# Retrospective study on the outcomes of Fassier-Duval nailing and osteotomy for the treatment of long bone fractures or deformities in the lower extremities in children with osteogenesis imperfecta

**DOI:** 10.3389/fsurg.2025.1706335

**Published:** 2025-12-04

**Authors:** Bin Jin, Yuan Chen, ChuanQing Bai, HaiLong Ma, JunChen Zhu

**Affiliations:** 1 Department of Pediatric Orthopedics, The First Affiliated Hospital of Anhui University of CM, Hefei, China; 2 Department of Orthopedics, Anhui Provincial Children’s Hospital, Hefei, China; 3 Department of Orthopedics, The Second Affiliated Hospital of Anhui University of CM, Hefei, China

**Keywords:** osteogenesis imperfecta, Fassier-Duval nail, osteotomy, fractures, deformities

## Abstract

**Background:**

Osteogenesis imperfecta (OI) is a rare hereditary disorder characterized by bone fragility and deformity, frequently requiring surgical intervention to restore limb alignment and stability. Traditional intramedullary fixation devices are limited in growing children. The Fassier-Duval (FD) nail, designed to elongate with growth, offers potential advantages when combined with corrective osteotomy. This study retrospectively evaluated the clinical outcomes of FD nail fixation with osteotomy for long bone fractures and deformities in children with OI.

**Methods:**

We retrospectively reviewed 33 pediatric OI patients (23 males, 10 females) who underwent FD nail implantation for 63 long bones of the lower extremities (42 femurs, 21 tibias) between December 2016 and September 2023. According to Sillence classification, 5 patients were type I, 18 type III, and 10 type IV. Thirty-two bones presented with acute fractures, 9 with isolated deformities, and 22 with fracture plus deformity. Radiographic outcomes, number of osteotomies, complication rates, revision surgeries, and functional results (PODCI and BAMF scores) were analyzed.

**Results:**

The mean age at initial surgery was 7 years, with a median follow-up of 55 months. The average time to bone union was 5.3 weeks (range, 4–7). For patients with deformities, mean preoperative coronal and sagittal angulations were 31.9° and 34.5°, corrected postoperatively to 0.9°and 1.1°, respectively. Thirteen revision surgeries were required, with an overall revision rate of 20.63%. Complications included refracture (*n* = 4) and nail failure (*n* = 9); no nonunion, nerve injury, or infection occurred. PODCI scores improved from 40.4 to 45.5, and BAMF scores from 3.1 to 6.1 (both *P* < 0.0001).

**Conclusions:**

FD nail fixation combined with osteotomy is effective for treating long bone fractures and deformities in children with OI. It provides reliable deformity correction, promotes rapid bone healing, and significantly improves motor function and quality of life, with acceptable complication and revision rates.

## 1 Introduction

Osteogenesis imperfecta (OI), also known as brittle bone disease, is a genetic disorder characterized by fragile bones that fracture easily with minimal force (1–4). The condition arises from mutations in genes encoding type I collagen, which is essential for maintaining bone strength (5, 6). The severity of osteogenesis imperfecta varies significantly, ranging from mild forms (rare fractures) to severe forms (frequent fractures, skeletal deformities, and other complications such as hearing loss and dental issues) (7). Children with OI experience a high incidence of fractures, often requiring surgical intervention to enhance bone stability and prevent deformities (8).

Traditionally, various intramedullary fixation devices, such as Rush nails and titanium elastic nails (TENs), have been used for fracture stabilization in children with OI (9, 10). While these methods provide stability, they have limitations: they require multiple surgeries to accommodate growth and development and are ineffective at preventing skeletal deformities in growing children (11).

To address these challenges, researchers developed intramedullary nail technologies like the Fassier-Duval (FD) nail. This technique offers the advantage of synchronous adjustment with the child's growth, significantly reducing the need for repeat surgeries (12, 13). The FD nail automatically lengthens to accommodate bone growth, maintaining continuous skeletal development without additional procedures (14). This characteristic makes it an ideal solution for treating fractures and deformities in children with brittle bone disease, particularly for lower limb fractures—where bone growth and proper alignment are critical for mobility.

Although several studies have previously reported the use of FD nails in patients with osteogenesis imperfecta, most existing series have focused primarily on early postoperative outcomes or complication profiles with relatively short follow-up durations. Moreover, few reports have systematically applied the Center of Rotation of Angulation (CORA)-based analysis to guide and quantitatively evaluate deformity correction.

In contrast, the present study provides a longer median follow-up period and incorporates CORA-based precision assessment to objectively evaluate alignment correction in both femoral and tibial procedures. By integrating these radiographic parameters with functional outcome measures (PODCI and BAMF scores), this study aims to offer a more comprehensive evaluation of the long-term structural and functional effectiveness of FD nail fixation in a large pediatric OI cohort.

## 2 Methods and materials

This retrospective study analyzed outpatient records from the Orthopedic Department of Anhui Children's Hospital between December 2016 and September 2023. A total of 63 lower limb long bones (42 femurs, 21 tibias) in 33 OI patients (23 males, 10 females) underwent lengthening intramedullary nailing and osteotomy correction surgery. The mean age at initial surgery was 7 years (range: 1–17 years). The median follow-up duration was 55 months (range: 26–104 months).

### 2.1 Inclusion and exclusion criteria

Inclusion criteria were as follows: (1) Clinically and genetically confirmed OI; (2) Fracture, severe deformity, or combined fracture-deformity in lower limb long bones (femur or tibia); (3) Requirement for FD telescopic intramedullary nail fixation, potentially combined with osteotomy correction. (4) Age range of 1–17 years. (5) Minimum postoperative follow-up of 12 months.

Exclusion criteria were: (1) Incomplete clinical or follow-up data. (2) Presence of neuromuscular disorders or other conditions potentially affecting motor function. (3) Concurrent infectious disease or severe scar adhesions at prior surgical sites. (4) Coagulation disorders.

### 2.2 Surgical approach

All surgeries were performed by a single surgeon (B.J.) and the same surgical team (H.L.M. and C.Q.B.). Procedures were conducted under C-arm fluoroscopy. Cefazolin was administered intravenously as antibiotic prophylaxis 30 min preoperatively, based on patient age and weight. Surgery was performed in the supine position under general anesthesia. Patients with non-deformed fractures underwent intramedullary fixation with FD nails alone, without osteotomy correction. Patients with deformed fractures or severe deformities without fractures received intramedullary fixation with FD nails combined with osteotomy correction. Osteotomy correction was performed for deformities with a CORA angle >20° or deformities affecting ambulation. All procedures in this study were based on the modified Sofield-Millar minimally invasive technique, performed under C-arm fluoroscopic guidance via a small incision with limited periosteal release and targeted osteotomy (15). No procedures posing fracture risks, such as blood pressure cuffs or tourniquets, were applied to patients.

#### 2.2.1 Femoral surgery

The surgical site was routinely prepared with antiseptic draping. For femoral osteotomy correction, the deformed segment was first identified using C-arm fluoroscopy, and the number of osteotomies was determined based on the severity of deformity. A 3–5 cm small incision was made slightly lateral to the greater trochanter apex. After inserting a guide wire through the femoral medullary canal to the deformity site, a small osteotomy incision was made at this location to correct the deformity. The guide wire was advanced until it reached the center of the distal femoral epiphysis, restoring normal anatomical structure to the femur. In cases without deformity, directly drill and insert the guide wire to enlarge the medullary cavity. Determine the appropriate FD nail length and diameter for the patient. Under C-arm fluoroscopy, first advance the male and female nails, ensuring the male nail threads extend beyond the distal epiphyseal plate and fixate 3–4 mm subchondrally at the center of the distal epiphysis. Lock the female nail at the greater trochanter.

#### 2.2.2 Tibial surgery

Prepare the surgical site with routine disinfection and draping. During tibial osteotomy correction, first identify the deformity area using C-arm fluoroscopy. With the knee maximally flexed, make a 2–3 cm incision below the patella and split the patellar tendon. Next, insert a guide wire at the tibial plateau slope through the tibial medullary canal to the deformity site. Make a small incision at this location to perform osteotomy correction until the guide wire passes through and reaches the center of the distal tibial epiphysis, restoring normal tibial anatomy. In cases without deformity, directly drill the guidewire into the medullary cavity to determine the appropriate FD nail length and diameter for the patient. Under C-arm fluoroscopy, advance the male and female nails so that the male nail threads extend beyond the distal epiphyseal plate and fixate 2–3 mm subchondrally at the center of the distal epiphysis, while the female nail locks into the proximal tibial epiphysis.

### 2.3 Postoperative management

All patients underwent plaster immobilization for 4–6 weeks postoperatively. For femoral procedures, a hip spica plaster was applied, while long-leg plaster were used for tibial procedures. Following plaster removal, patients initiated joint rehabilitation and weight-bearing exercises in water therapy, with full weight-bearing permitted at 8 weeks.

### 2.4 Data collection

The following data were collected from patient records: OI severity and classification assessed using the Sillence criteria (1). Imaging studies, number of osteotomies, postoperative fractures, and time to continuous callus formation at osteotomy sites. Intraoperative and postoperative complications, with incidence rates calculated. Revision surgery frequency and time interval since initial surgery, with revision rate calculated. Coronal and sagittal CORA angles were measured preoperatively and at 1 year postoperatively (First, identify the site of deformity by examining full-length x-rays of the lower limbs in coronal and sagittal planes, marking the anatomical axes of the proximal and distal bones. Next, extend these two axes to locate their intersection point, which is the CORA point. Then, measure the angle formed between each axis and the CORA point—this is the CORA angle. A larger angle indicates more severe deformity. All CORA angle measurements are performed by an experienced pediatric orthopedic surgeon.) (16, 17). Preoperative and 1-year postoperative PODCI (Pediatric Outcome Data Collection Instrument) scores were recorded (18). The pediatric form (2 to 10 years) is completed by parents. The adolescent form (11 to 18 years) is answered by parents (Parent-Report) and adolescents (Self-Report). The parent version comprises 86 items, while the adolescent self-report version includes 83 items. Each scale's results are converted through standardization to a range of 0–100 points, where 0 represents the lowest score and 100 represents the highest (18, 19). Preoperative and 1-year postoperative BAMF (Brief Assessment of Mobility Function) scores were also documented (20). Rapidly assess children's gross motor, fine motor, and oral motor skills using a 10-point rating scale. Evaluators record the highest score based on the child's performance during natural activities, including rolling over, sitting, walking, and other tasks.

### 2.5 Statistical analysis

Data processing and analysis were performed using the Statistical Package for the Social Sciences (version 26.5; SPSS Inc., Chicago, IL, USA). Descriptive statistics presented quantitative variables as median with minimum and maximum values, and qualitative variables as frequency and percentage. For normally distributed data, between-group comparisons were performed using independent samples *t*-tests, and multi-group comparisons were conducted using one-way analysis of variance (ANOVA). *post-hoc* pairwise comparisons performed using Tukey correction to control for multiple testing. For data not meeting normality or homogeneity of variance assumptions, significant differences between groups were assessed using the Mann–Whitney *U*-test. The significance level for two-tailed tests was set at *P* < 0.05.

## 3 Results

### 3.1 General findings

Patient severity of OI was assessed using the Sillence classification: 5 patients were Type I, 18 were Type III, and 10 were Type IV. FD nails were used for fixation in all 63 lower limb long bones (42 femurs and 21 tibias) among the 33 OI patients. 20 patients underwent surgery involving osteotomy for deformity correction, 33 patients received FD nailing for fracture fixation, and 9 patients underwent prophylactic fixation to prevent impending fractures in areas of severe deformity or cortical thinning. Among these 63 lower limb long bones: 32 cases involved fractures without deformity, 9 cases involved severe deformity without fracture, 22 cases involved fractures with deformity. For the 13 revision surgeries, the mean interval from the initial surgery was 23.7 months (range: 4–61 months). Among these, the 8 femoral revision cases had an average interval of 37.3 months (range: 6–61 months), while the 5 tibial revision cases had an average interval of 19 months (range: 4–42 months). The revision rate for femoral procedures was 19.05%, for tibial procedures 23.81%, and the overall revision rate was 20.63%.

### 3.2 Comparison of radiographic outcomes

**Figure 1:**
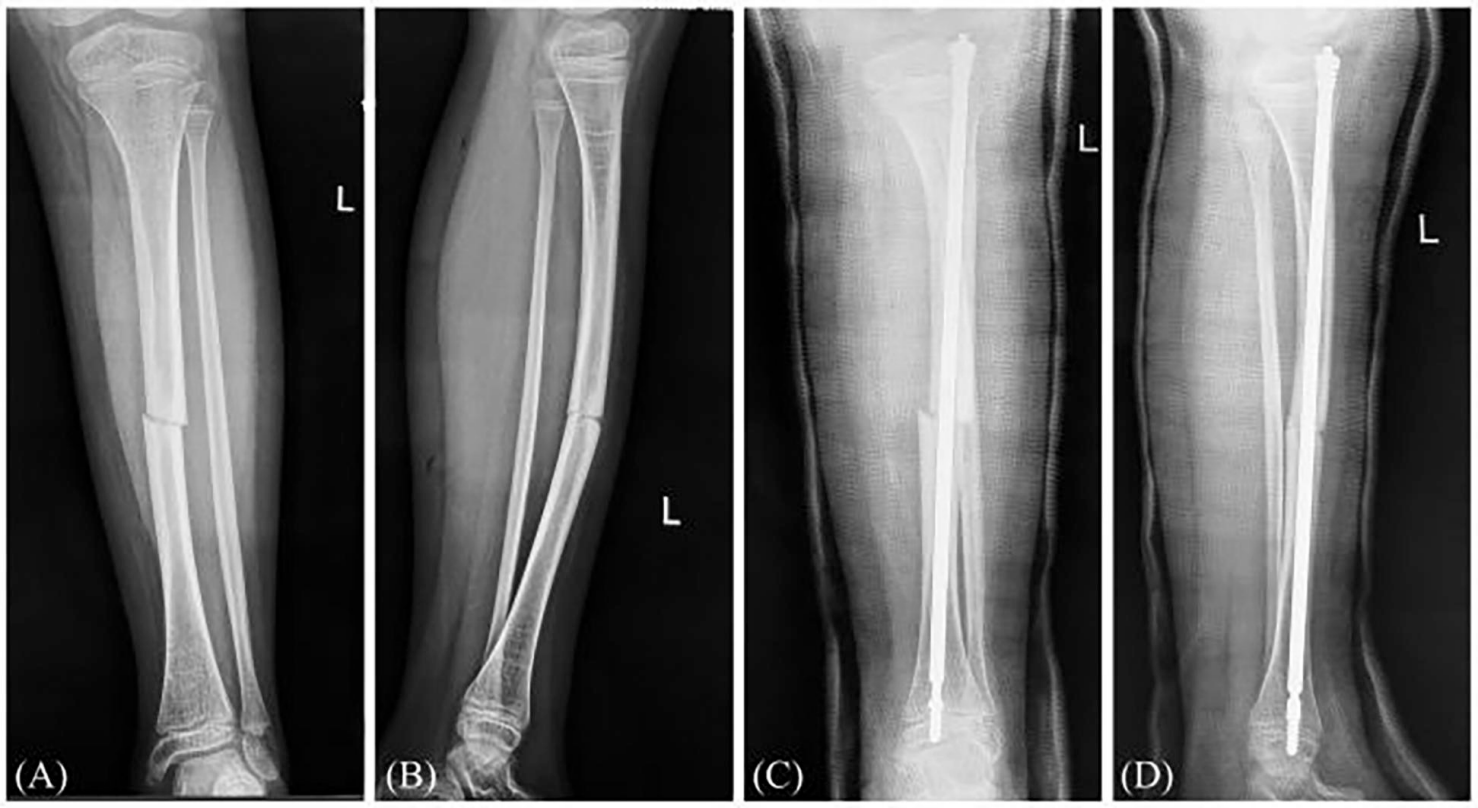
Radiographs of the left tibia in a 9-year-old patient. **(A,B)** Anteroposterior and lateral views showing a diaphyseal fracture of the left tibia. **(C,D)** Immediate postoperative anteroposterior and lateral views following FD nail internal fixation of the left tibia.

**Figure 2:**
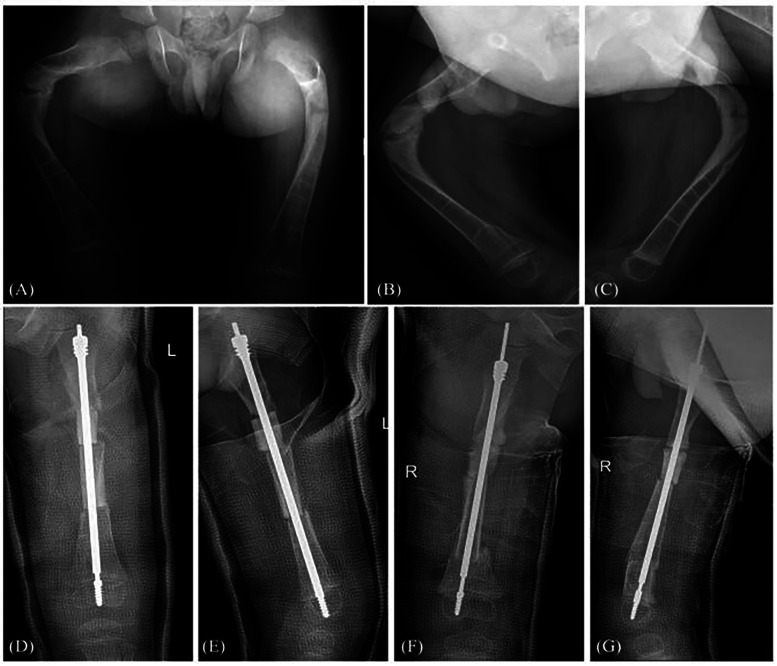
Radiographs of the bilateral femurs in a 3-year-old patient. **(A–C)** Show anteroposterior and lateral views of bilateral femoral fractures and deformity. **(D,E)** Show immediate postoperative anteroposterior and lateral views of the left femur after FD nail internal fixation and osteotomy correction. **(F,G)** Show immediate postoperative anteroposterior and lateral views of the right femur after FD nail internal fixation and osteotomy correction.

Postoperative follow-up radiographs revealed that the mean time to achieve continuous callus formation at the surgical site in 63 lower limb long bone cases was 5.3 weeks (range: 4–7 weeks). For 9 patients with severe deformity (no fracture) and 22 patients with acute fracture combined with deformity, analysis of immediate preoperative radiographs revealed an average angular deviation of 31.9° (range 2–89°) in the coronal plane and 34.5° (range 9–91°) in the sagittal plane. Postoperative radiographs demonstrated correction of the mean coronal angle to 0.9° (range 0–2°) and the mean sagittal angle to 1.1° (range 0–2°), with an average of 2 osteotomies (range 1–3). Preoperative and immediate postoperative radiographs of the tibia are shown in Figure 1. Preoperative and immediate postoperative radiographs of the femur are shown in Figure 2.

### 3.3 Comparison of PODCI and BAMF scores

PODCI scores showed a preoperative mean of 40.4 points (range 27–75) and a mean of 45.5 points (range 34–81) at follow-up completion. The BAMF scores showed a preoperative mean of 3.1 points (range 2–5) and a postoperative mean of 6.1 points (range 5–8) at follow-up. The differences in PODCI and BAMF scores between preoperative and postoperative follow-up were statistically significant (*P* < 0.0001; *P* < 0.0001) (Table 1).

**Table 1:** Patient preoperative and 1-year postoperative PODCI and BAMF scores.

Time	PODCI	BMAF
Preoperative	40.4	3.1
1 year postoperative	45.5	6.1
*t*	34.86	95.5
*P*	<0.0001	<0.0001

### 3.4 Incidence and management of complications

**Figure 3:**
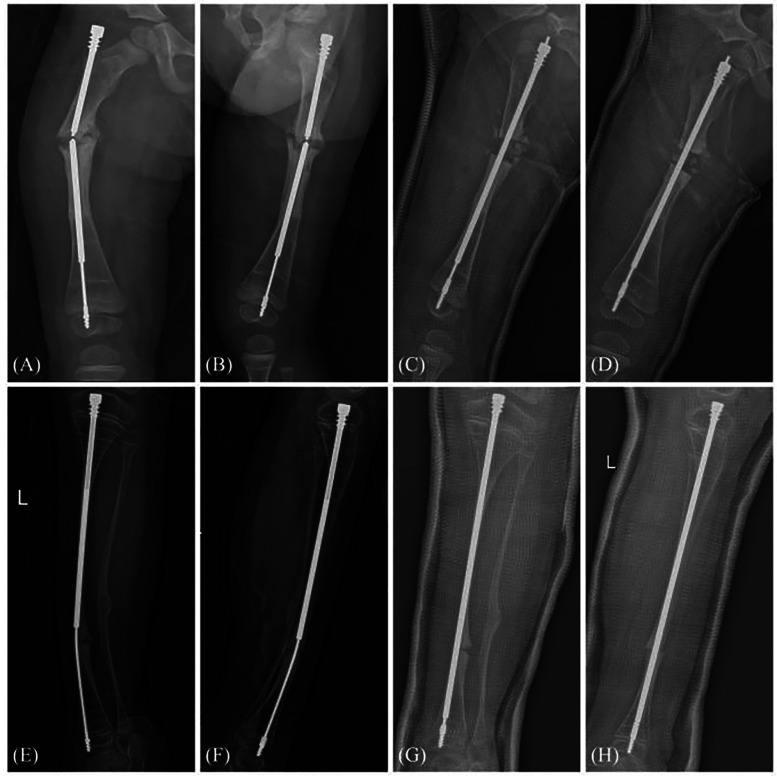
Radiographs showing complications at 6 months postoperatively in the right femur of a 2-year-old patient. **(A,B)** Anteroposterior and lateral views demonstrating fracture of the right femoral FD nail. **(C,D)** Immediate postoperative anteroposterior and lateral views following revision surgery for right femoral FD nail fracture. X-ray showing complications at 39 months postoperatively in an 8-year-old patient. **(E,F)** Anteroposterior and lateral views showing bending of the left tibial FD nail. **(G,H)** Anteroposterior and lateral views immediately after revision surgery for bending of the left tibial FD nail. Both patients received replacement FD nails.

Among the 63 lower limb procedures performed, the overall complication rate was 20.63% (13/63), with rates of 19.05% (8/42) for femoral surgeries and 23.81% (5/21) for tibial surgeries (Table 2). No cases of non-union, infection, or nerve injury were observed during the follow-up period, indicating that the FD nailing procedure, combined with osteotomy, provided a stable biological environment conducive to bone healing. A total of four cases of postoperative refracture (6.35%) were recorded—three involving the femur and one involving the tibia. All refractures occurred following trauma within 6–18 months postoperatively. FD nail-related complications were the most frequent, accounting for nine cases (14.29%), including nail dislodgement, bending, or mechanical failure of the telescoping components. Among these, five occurred in femoral nails and four in tibial nails. Nail displacement typically developed within 12–6 months post-surgery and was associated with premature weight-bearing or rapid growth in the metaphyseal region. In most cases, the failures were managed by revision surgery involving nail replacement or adjustment (Figure 3).

**Table 2:** Complications seen in our patients.

Complications	Femur (*n* = 42)	Tibia (*n* = 21)	Total (*n* = 63)
Non-union	0	0	0
Refracture	3	1	4
Infection	0	0	0
Nerve damage	0	0	0
FD nail invalid	5	4	9
Total	8	5	13
Rate/%	19.05%	23.81%	20.63%

## 4 Discussion

This study aimed to evaluate the clinical efficacy of FD nails combined with osteotomy correction in treating long bone fractures and deformities of the lower limbs in children with OI. Results demonstrated that FD nails with osteotomy reliably corrected coronal and sagittal plane deformities, accelerated callus formation, and significantly improved PODCI and BAMF functional scores. Notably, the overall complication rate and revision rate remained within acceptable ranges, with no cases of nonunion, infection, or nerve injury reported. These findings highlight the FD nail's advantage in providing long-term stable fixation while accommodating pediatric skeletal growth. This surgical approach also substantially enhances patients' quality of life. By improving lower limb function, children can perform daily activities such as walking and stair climbing with greater ease. For example, children who required wheelchairs or crutches for mobility before surgery can walk independently afterward, with greatly enhanced self-care abilities and a qualitative improvement in their quality of life.

This study precisely measured the CORA angle preoperatively to formulate orthopedic correction plans, enabling more targeted design of osteotomy sites and correction angles. This significantly improved the accuracy of postoperative alignment restoration. Results revealed preoperative mean coronal angles of 31.9° and sagittal angles of 34.5° among pediatric patients. Immediate postoperative radiographs demonstrated mean residual angles of only 0.9° in the coronal plane and 1.1° in the sagittal plane, indicating near-complete correction of deformity. This precise orthopedic correction based on the CORA principle not only optimized surgical outcomes but also reduced the risk of complications arising from undercorrection or overcorrection.

Compared to previous studies using traditional intramedullary fixation devices (such as Rush nails or elastic titanium nails), this study demonstrated a lower recurrence rate of deformity and fewer revision surgeries, consistent with the theoretical advantages of FD nails (21). Previous studies have also indicated that refractures and implant failure are common issues. The types of complications in this study were similar, but their incidence rates were relatively lower, possibly related to the inherent bone fragility of OI and the technical difficulty of nail placement (22). Gamble et al. reported a complication rate of 55% with non-extensible nails and a complication rate of 69% with Bailey-Dubow nails (23). Compared to the reported 69% complication rate, the 20.63% rate in this study is more favorable, likely due to strict patient selection, standardized surgical procedures, and systematic postoperative rehabilitation strategies.

Multiple previous studies have explored the application of FD nails in children with OI (24, 25). Birke et al. reported that FD nails significantly reduce the recurrence of deformity and re-fracture rates, while decreasing the need for reoperation (26). Our findings are broadly consistent with these reports, demonstrating the FD nail's advantages in improving lower limb alignment, promoting bony union, and enhancing function (27). However, a notable difference from some studies is our relatively low overall revision rate (20.63%), whereas certain literature reports revision rates exceeding 30% (28). Furthermore, the relatively extended follow-up period in this study provides more representative assessments of efficacy and complications. We acknowledge that direct comparison of numerical outcomes across single-center studies has inherent limitations, as such differences may reflect institutional factors—such as surgical expertise, patient selection criteria, or postoperative care protocols—rather than the intrinsic superiority of the implant itself.

The following points may explain the results of this study: First, preoperative optimization played an important role. All patients received standardized bisphosphonate therapy and nutritional assessment before surgery, which improved bone mineral density and reduced intraoperative fragility. Second, refinements in surgical planning and technique contributed to improved mechanical stability. All deformity corrections were guided by CORA-based angular analysis, allowing precise multi-planar osteotomy correction and accurate intramedullary alignment. Centralized positioning of the FD nail and secure fixation of both telescopic components minimized the risk of migration, bending, or mechanical failure. Third, our postoperative management protocol was highly structured. Patients with femoral procedures were immobilized with hip spica casts, while tibial cases used long-leg casts for 4–6 weeks, followed by water-based rehabilitation and gradual weight-bearing after 8 weeks. This controlled progression protected the surgical construct during early healing while maintaining joint mobility. Finally, our long median follow-up period (55 months) allowed continuous monitoring and early intervention in minor complications, preventing their progression to implant failure or refracture.

From a consistency perspective, this study reaffirms the efficacy and safety of FD nails in correcting lower limb deformities and treating fractures in children with osteogenesis imperfecta. Unlike previous studies reporting only radiographic indicators or complication rates, this study expanded its design to include functional outcome assessments. The use of the PODCI and BAMF scales systematically reflected improvements in patients' quality of life and motor function, thereby providing a more comprehensive evaluation of treatment efficacy. Furthermore, this study had a larger sample size than some single-center studies and included patients with Sillence Types I, III, and IV deformities, enhancing its clinical applicability. More importantly, this study features an extended follow-up period (median 55 months), providing long-term data support. This is crucial for validating the durability of surgical outcomes and mitigating short-term result biases.

Surgery may induce multiple complications (29, 30). Screw migration is a common complication potentially caused by premature weight-bearing, vigorous activity, inadequate FD screw fixation, or excessive screw thread depth (31). Recurrence of fracture is another significant concern, potentially arising from the inherent fragility of pediatric bones or inadequate postoperative rehabilitation protocols. Infection represents a further risk, as surgical sites may become contaminated with bacteria, leading to impaired wound healing or severe complications like osteomyelitis. These complications not only compromise surgical outcomes but may also inflict additional suffering and financial burdens on patients while prolonging recovery periods.

## 5 Conclusions and outlook

### 5.1 Conclusions

Through retrospective analysis of clinical data from pediatric osteogenesis imperfecta patients treated with FD nail internal fixation combined with osteotomy correction surgery, this study reached the following primary conclusions. This surgical approach demonstrated significant efficacy in treating lower limb long bone fractures or deformities in pediatric osteogenesis imperfecta, effectively correcting lower limb deformities and restoring normal lower limb alignment. Postoperative improvement in lower limb bowing and angulation deformities laid a solid foundation for functional recovery. Regarding fracture healing, the procedure promoted favorable outcomes, with most fracture sites meeting clinical healing criteria within the specified postoperative timeframe and ultimately achieving bony union. This approach effectively reduced the risk of nonunion or delayed union.

Regarding functional improvement, postoperative patients demonstrated markedly enhanced limb function and significantly increased capacity for activities of daily living. Comparisons of PODCI and BAMF scores revealed substantial gains in self-care, transfer, and walking dimensions, indicating markedly improved quality of life. However, the procedure carries certain complication risks, with nail displacement and re-fracture being relatively common. Most complications were effectively resolved through conservative treatment or reoperation.

### 5.2 Outlook

This study has certain limitations. The relatively small sample size may limit the generalizability of findings and prevent a comprehensive reflection of surgical outcomes across different patient types. The relatively short study duration precludes in-depth investigation of long-term effects, such as the impact on skeletal development and limb function in adulthood. Future studies should expand sample sizes to include a broader range of patient types and disease severities, conducting multicenter, large-scale investigations to enhance the reliability and generalizability of findings.

Extended follow-up periods are necessary to thoroughly evaluate the long-term effects of surgery on skeletal development and limb function, as well as the long-term incidence of complications. Concurrently, with ongoing advancements in medical technology, new surgical techniques and materials should be actively explored. This includes further refining the design and performance of FD nails to enhance surgical outcomes and reduce complication rates. Research into the pathogenesis of osteogenesis imperfecta should be intensified, with efforts to identify more effective treatments at the genetic level. This will enable the provision of more comprehensive and effective therapeutic strategies for affected children.

## Data Availability

The raw data supporting the conclusions of this article will be made available by the authors, without undue reservation.
